# Effect of Lactobacteria on Bioactive Peptides and Their Sequence Identification in Mature Cheese

**DOI:** 10.3390/microorganisms10102068

**Published:** 2022-10-19

**Authors:** Marina Kurbanova, Roman Voroshilin, Oksana Kozlova, Victor Atuchin

**Affiliations:** 1 Department of Animal Products Technology, Technological Institute of Food Industry, Kemerovo State University, Stroiteley Ave., 47., 650056 Kemerovo, Russia; 2 Department of Biotechnology, Technological Institute of Food Industry, Kemerovo State University, Stroiteley Ave., 47., 650056 Kemerovo, Russia; 3 Laboratory of Optical Materials and Structures, Institute of Semiconductor Physics, 630090 Novosibirsk, Russia; 4 Research and Development Department, Kemerovo State University, 650000 Kemerovo, Russia; 5 Department of Industrial Machinery Design, Novosibirsk State Technical University, 630073 Novosibirsk, Russia; 6 R&D Center “Advanced Electronic Technologies”, Tomsk State University, 634034 Tomsk, Russia

**Keywords:** starter cultures, bioactive peptides, semi-hard cheeses, *Lactococcus lactis* subsp. *lactis*, *Lactococcus lactis* subsp. *cremoris*, *Lacticaseibacillus casei*

## Abstract

An in silico study that featured the effect of starter cultures on the bioactivity and other health benefits of peptides in semi-hard cheese is presented in this contribution. Model Caciotta-type cheese samples were obtained in laboratory conditions in two variations. Sample A included starter cultures of *Lactococcus lactis* subsp. *lactis* and *Lactococcus lactis* subsp. *cremoris*. Sample B included starter cultures of *Lactococcus lactis* subsp. *lactis, Lactococcus lactis* subsp. *cremoris,* and a culture of lactobacilli *Lacticaseibacillus casei*. The in silico method showed that the peptides inhibited angiotensin-converting enzymes (ACE) and ipeptidyl peptidase IV (DPP-4), as well as possessed antioxidant properties. *Lactococcus lactis* subsp. *lactis* and *Lactococcus lactis* subsp. *cremoris* had a greater effect on the formation of bioactive peptides.

## 1. Introduction

Modern foods not only provide the body with nutrients and satisfy hunger, but also improve physical and mental well-being and prevent nutrition-related diseases [1,2,3,4,5,6,7]. If consumed regularly, antioxidants improve human health and life quality, inhibit aging, and prevent cancer, cardiovascular diseases, neurological disorders, etc. [8,9,10,11,12,13,14,15,16,17,18,19,20].

Dairy products are consumed in all parts of the world, and the dairy sector is the most prominent market of functional food with global prospects. Safe and live microbes can be fermented with specially cultivated strains and obtain health-promoting properties that reduce the risk of certain diseases if these functional products are part of one’s daily diet. Cheese is one of such dairy products [21,22,23,24,25]. Cheese is a fresh or mature dairy product that is obtained by coagulating milk with enzymes, microorganisms, acids, etc. This product is an integral part of almost all traditional cuisines, and its historical role in human diet can hardly be overestimated. Cheese is easy to digest and rich in nutrients, which makes it an important and versatile source of proteins, short-chain fatty acids, vitamins, and minerals, depending on the region [26,27,28,29,30].

Biochemical reactions that occur in semi-hard and hard cheeses during ripening shape their sensory profile. These reactions result from the metabolism of lactic acid bacteria (LAB) introduced as starter cultures during the production process, as well as bacteria that are initially present in raw milk or may enter it from the environment. The nutrient medium for the cheese microbiota in ripening cheese includes short peptides or amino acids of protein molecules, citrates, lactates, bacteriolysis products of starter cultures of LAB, and free fatty acids [31,32].

Food-derived bioactive peptides are becoming more and more popular as the global consumer gets more aware of their specific properties that help strengthen the immune status. Bioactive peptides are relatively small fragments of dietary proteins. As a rule, they consist of 2–20 amino acid residues. Bioactive peptides can be ligands, and therefore they can target the immune, cardiovascular, digestive, and endocrine systems [33,34,35]. Some food-derived peptides have antioxidant, immunomodulatory, antihypertensive, anticancer, anti-inflammatory, antimicrobial, opioid, and hypocholesterolemic properties. They can modulate intestinal microbiome and prevent diabetes and chelate metals [36,37,38]. The biological activity of peptides depends mainly on their amino acid composition, sequence, length, and charge of peptides [39,40,41].

Modern science knows hundreds of peptides with different biological activities identified and isolated from various food sources, e.g., milk, whey, eggs, fish, rice, soybeans, peanuts, chickpeas, corn, and some algae [42]. However, only a few peptides have become functional nutraceuticals that are used in food production. Only milk and fish bioactive peptides seem to have established a relatively high presence in the current food ingredient market. Antioxidant peptides usually contain hydrophobic amino acids and histidine, phenylalanine, tryptophan, or tyrosine residues [43,44].

Bioactive peptides are obtained from milk or other food proteins during proteolysis under the action of proteolytic enzymes and microbial fermentation. They are currently of great interest to scientists [45,46,47]. Cheeses are purely protein products, which makes them the main precursors of bioactive peptides, as well as milk protein hydrolysates. Bioactive peptides are organic molecules with a potential biological activity that can affect certain body functions and human health in general. Bioactive peptides can serve as alternative preventive treatments against various metabolic diseases because they have a broad-spectrum and biospecific activity. Moreover, they are hypoallergenic and structurally diverse. Depending on the amino acid sequence, composition, length, and charge, active peptides can exhibit an impressive array of biological properties. They possess antioxidant, antimicrobial, immunomodulatory, anticancer, antihypertensive, anti-inflammatory, opiate, and antilipidemic activities that improve the cardiovascular, gastrointestinal, immune, and endocrine systems. They do not accumulate in the body and quickly degrade in the environment. They usually contain 2–20 amino acid residues and are encoded in the primary structure of animal and plant proteins in an inactive form [48,49,50].

Our research objective was to study the effect of starter cultures on the bioactivity and other health benefits of peptides in semi-hard cheeses using the in silico method. The experiment featured ninety-day-old model Caciotta-type cheeses with *Lactococcus lactis* subsp. *lactis* (*Lc. lactis* subsp. *lactis*), *Lactococcus lactis* subsp. *cremoris* (*Lc. lactis* subsp. *cremoris*) and *Lacticaseibacillus casei* (*Lac. casei*). They were tested for chemical composition, peptide sequence, and molecular weight of peptides. The in silico analysis featured the bioactivity, structure, and hydrophobic and hydrophilic properties of the identified peptides. The results obtained made it possible to identify the effect of starter cultures on the bioactivity and other properties of the semi-hard cheese peptides.

## 2. Materials and Methods

The model cheese samples of the Caciotta type were produced in laboratory conditions from Jersey cow milk.

### 2.1. Model Caciotta-Type Cheese Production

The standard Caciotta cheese has a protein/fat ratio of 0.92. Table 1 presents the main technological parameters applied in the laboratory production of of cheese Caciotta.

**Table 1:** Main technological parameters for the production of laboratory samples of cheese Caciotta.

Starting Components		Basic Cheese-Making Parameters	
cow milk	10 L	Pasteurization milk	64 °C, 30 min
annatto (for winter milk) *	5–6 drops	Inoculation (fermentation)	34 °C, 10 min
* prefabricated bacterial mesophilic culture (*Lc. lactis* subsp. *lactis* V-1568; *Lc. lactis* subsp. *cremoris* V-1569) **(control cheese A)** (*National Bioresource Center Russian Collection of Industrial Microorganisms (VKPM*))	150 g (0.5% of the milk volume	Flocculation multiplier	3
** prefabricated bacterial mesophilic culture (*Lc. lactis* subsp. *lactis* V-1568; *Lc. lactis* subsp. *cremoris* *V*-1569; *Lac. casei* V-9227) **(experimental cheese B)** (*National Bioresource Center Russian Collection of Industrial Microorganisms (VKPM*))	100 g (50/50, respectively)	Cheese cube size	1.0 cm
calcium chloride (10–20% solution CaCl_2_) in an aqueous solution (for pasteurized and winter milk)	10 mL	Granular curd heating temperature	45 °C
milk-clotting enzyme *(“Carlina” (composition: 90% rennet chymosin, 10% pepsin; manufacturer: Danisco France SAS, France)*	0.35 g (in the amount necessary for 12–15 min of flocculation time)	Stuffature (the cheese head was turned eight times);	1.5 h at 45 °C
		Development at room temperature ≥ 22 °C	4 h
		Maturation time (see final product in Figure 1).	90 days

* prefabricated bacterial mesophilic culture **(control cheese A)** (*Lc. lactis* subsp. *lactis* V-1568; *Lc. lactis* subsp. *cremoris* V-1569) (*National Bioresource Center Russian Collection of Industrial Microorganisms (VKPM*)). ** prefabricated bacterial culture **(experimental cheese B)** (*Lc. lactis* subsp. *lactis* V-1568; *Lc. lactis* subsp. *cremoris* *V*-1569; *Lac. casei* V-9227) (*National Bioresource Center Russian Collection of Industrial Microorganisms (VKPM*)).

**Figure 1:**
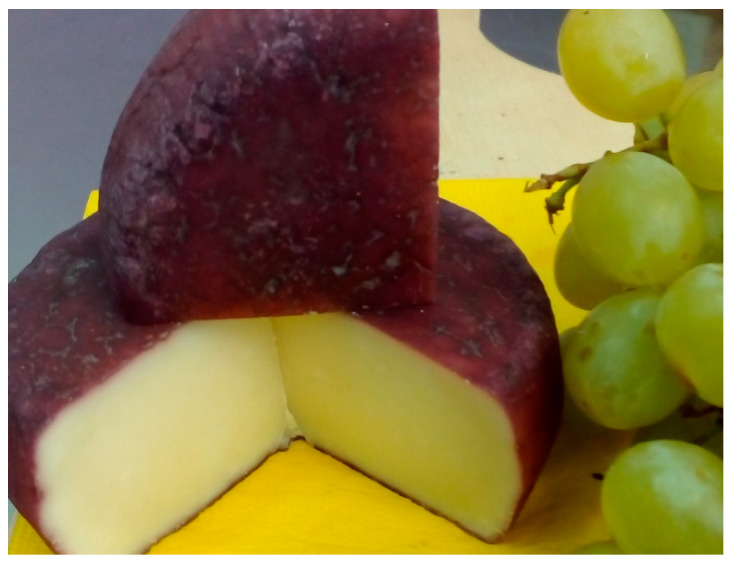
Mature Caciotta-type cheese.

### 2.2. Micrographs of the Lactobacilli

Micrographs of the lactobacilli were obtained using a scanning electron microscope with systems for an energy-dispersive microanalysis, a wave dispersion microanalysis, and a Nova NanoSEM 450 backscattered electron diffraction analysis system (Czech Republic) [51].

The test sample with a volume of about 1–5 g was placed in a liquid dispersion module (volume 500 mL). The measurement started automatically as soon as the absorbance value reached the specified value.

Measurement parameters:

– Type of measurement—according to the Fraunhofer method;
– Measurement range—from 0.1 μm to 1021.87 μm;
– Resolution—102 channels;
– Absorption—10.00%;
– Measurement duration—90 scans.

### 2.3. Protein Analyses

The protein mass fraction was determined using a Rapid N Cube total nitrogen (protein) analyzer by the Dumas method: after burning the sample, the total nitrogen was registered by a thermal conductivity detector [52].

### 2.4. The Molecular Weight Distribution

The molecular weight distribution was determined by sodium dodecyl sulfate-polyacrylamide gel electrophoresis (SDS-Na) [51]. Proteins were fractioned in denaturing polyacrylamide gel (separating 12% and focusing 4%) with 0.1% of SDSNa. The electrophoresis procedure was performed at a single buffer with the addition of 0.1% SDS-Na at 15 mA. The gel was dyed with 0.2% of Kumassi R-250 dye (prepared in glacial acetic acid) and then rinsed three times with distilled water. Gel visualization and analysis were performed using the Gel Doc XR+ Gel Documentation System. The molecular weight was calculated using the Peptide Mass Calculator (http://rna.rega.kuleuven.be/masspec/pepcalc.htm (accessed on 1 June 2022)).

### 2.5. Amino Acid Analyses

The amino acid sequence of the hydrolysate peptides was determined by the method of matrix-activated laser desorption/ionization on a MALDI Biotyper (Bruker), equipped with a UV laser (Nd) in the positive ion mode using a reflectron; the accuracy of the measured monoisotopic masses after the additional calibration by trypsin autolysis peaks was 0.005% (50 ppm). The spectra were obtained in the mass range of 600–5000 *m*/*z*, choosing the laser power that was optimal for reaching the best resolution. To obtain the fragmentation spectra, the tandem device mode was used; the measurement accuracy of fragment ions was no lower than 1 Da. [53].

### 2.6. Biological Activity of Peptides In Silico

The peptide bioactivity was assessed in silico using the PeptideRanker online server (http://distilldeep.ucd.ie/PeptideRanker/ (accessed on 5 June 2022)), which ranks peptides according to their potential biological activity [54].

### 2.7. Visualization of Dihedral Amino-Acid Angles

The visualization of the dihedral angles φ against ψ of amino acid residues in the protein structure was performed using the SWISS-MODEL resource (https://swissmodel.expasy.org/interactive (accessed on 8 June 2022).) according to the simulated Ramachandran Maps [55].

### 2.8. D protein Structure Modeling

The structure modeling stage employed the SWISS-MODEL service (https://swissmodel.expasy.org/interactive (accessed on 10 June 2022)), which includes the SWISS-MODEL repository and the SWISS-MODEL interactive workspace. This automated protein structure homology modeling platform generates 3D protein models using a comparative approach and a database of existing models for key reference proteomes based on UniProtKB [55]. 

### 2.9. Modeling the Structure of Peptides

The structure of the peptides was modeled using the PepDraw online tool (http://www2.tulane.edu/~biochem/WW/PepDraw/ (accessed on 15 June 2022)). The MBPDB database of bioactive milk peptides made it possible to identify and determine the protein and the properties of the peptides [56].

### 2.10. Hydrophobicity and Hydrophilicity of Proteins

The hydrophobicity and hydrophilicity of proteins were predicted based on the ProtScale online service (https://web.expasy.org/protscale/ (accessed on 19 June 2022)). This online service calculates and constructs two-dimensional graphs for the protein profile selected by any amino acid scale. It determines the amino acid scale by the numerical value assigned to each type of amino acid. The method uses the Kite and Doolittle scale that features individual values for 20 amino acids: Ala: 1.800, Arg: −4.500, Asn: −3.500, Asp: −3.500, Cys: 2.500, Gln: −3.500, Glu: −3.500, Gly: −0.400, His: −3.200, Ile: 4.500, Leu: 3.800, Lys: −3.900, Met: 1.900, Phe: 2.800, Pro: −1.600, Ser: −0.800, Thr: −0.700, Trp: −0.900, Tyr: −1.300, Val: 4.200, −3.500, −3.500, −0.490. When assessing the topology of a protein and its hydrophobicity by the Kite and Doolittle scale, the graph peaks that are greater than zero characterize the hydrophobic region, and those below zero describe the hydrophilic region [57].

## 3. Results

The model cheese samples were synthesized according to the technology described in Section 2.1. The model cheeses (250 ± 20 g) ripened at 12–14 °C and relative humidity of 80–85%. Their chemical composition and active acidity were determined on day 90 (Table 2).

It is shown in Table 1 that the chemical composition of the model cheeses was almost the same. However, the active acidity was lower in the experimental samples with *Lac. casei* (0.5% + 0.5%). The biochemical properties of LAB include acid formation energy, limiting acidity, ability to ferment citric acid salts, curd quality, proteolytic activity, etc. [24]. Lactic acid streptococci have different activities. *L. lactis* was the first microorganism isolated in pure culture (in 1873 by Lister). *L. lactis* subsp. lactis is homofermentative bacteria. During ripening, these bacteria ferment glucose via the fructose bisphosphate pathway also known as the Embden-Meyerhof-Parnas (E.M.P.) pathway, which is similar to that of alcohol. Pyruvate, however, does not decarboxylate to acetaldehyde, like in alcoholic fermentation: it is used directly as an electron (hydrogen) acceptor. D-lactate dehydrogenase in LAB marks the formation of D(–)-lactic acid, while L-lactate dehydrogenase marks L(+)-lactic acid. DL-lactic acid is determined by the synthesis of two lactate dehydrogenases of different stereospecificity accompanied by the L(+)-lactic acid formation. They are strong acid formers and exhibit proteolytic activity during cheese ripening [58,59,60,61].

**Figure 2:**
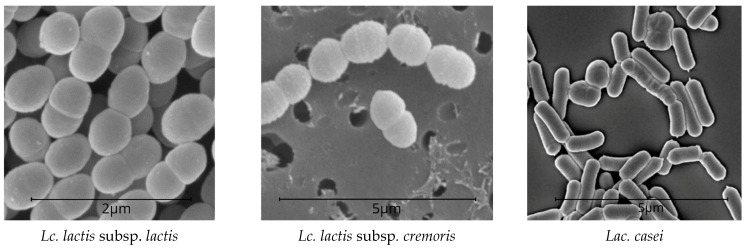
LAB SEM patterns (Nova NanoSEM-450).

Unlike *L. cremoris* neither ferment maltose and dextrin nor de-aminates arginine. At low cultivation temperatures (15–20 °C), some strains form a significant number of volatile acids. The energy of acid formation in *L. cremoris* is weaker than in *L. lactis*. *Lac. casei* is homofermentative and ferments lactose, releasing mostly lactic acid; however, these bacteria develop slowly in milk. *Lac. casei* has prominent saccharolytic properties. It ferments fructose, galactose, mannitol, mannose, raffinose, ribose, salicin, sorbitol, trehalose, esculin, etc. Glucose is fermented without gas formation. Lactobacilli produce a number of hydrolytic enzymes, e.g., lactase, which breaks down lactose (milk sugar) and prevents lactase deficiency [62,63]. *Lac. casei* is found in various cheeses, especially at the late ripening stages. *Lac. casei* can form chains with different numbers of cells and produce gas from sodium citrate [64]. LAB is shown in Figure 2.

**Figure 3:**
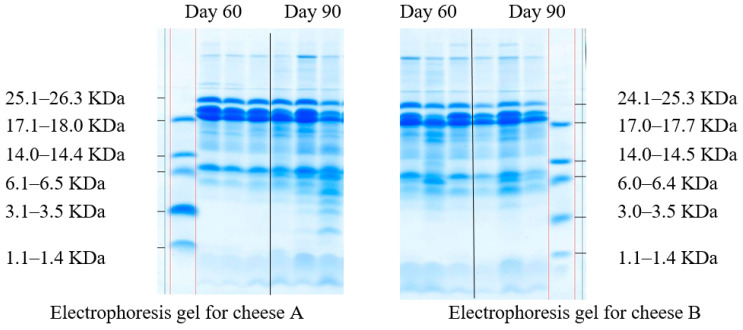
Molecular weight distribution of casein fractions.

The change in casein fractions during the proteolysis of milk proteins was determined in the model cheese samples by the electrophoresis in polyacrylamide gel (Figure 3).

The proteolytic activity during cheese ripening depends on several factors, such as type of coagulant, native milk microbiota and starter cultures, residual effect of the coagulant and native milk proteases, which can be affected by the moisture content in the cheese, its temperature, and relative humidity, ripening conditions, etc. Electrophoresis is one of the most common observation methods for cheese ripening. It can detect various casein fractions and protein breakdown products throughout the whole ripening process [65]. Fractions of α-casein are more susceptible to proteolysis, while degradation of β-casein occurs much less frequently [66]. Electrophoresis methods separate proteins by molecular weight and compare the staining intensity of polypeptide chains in the polyacrylamide gel.

Clear casein bands with different levels of hydrolysis throughout the ripening period, which indicates that the process involved several factors are shown in Figure 3. Although in Figure 3 are clear bands of various casein fractions, the fractions with the lowest molecular weight prevailed both on day 60 and day 90 of ripening. All samples demonstrated peptides with a molecular weight of 1.1–14.5 KDa. As for the sensory evaluation, cheese B had a more pronounced taste and aroma on day 90 due to *Lac.* *casei* in its starter culture. After 90 days of ripening, both cheeses were tested for peptide sequences and their bioactivity (Table 3).

**Figure 4:**
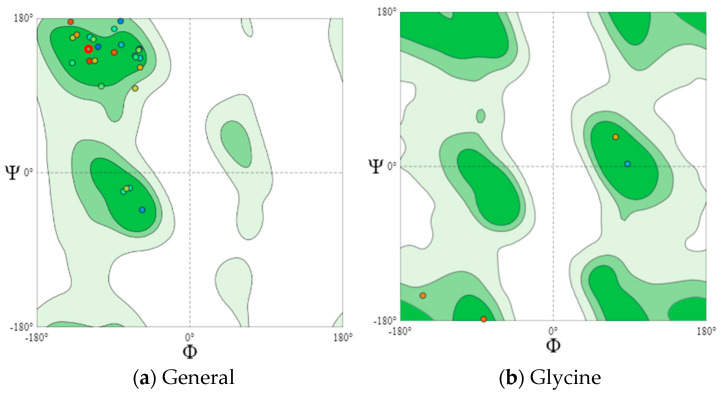

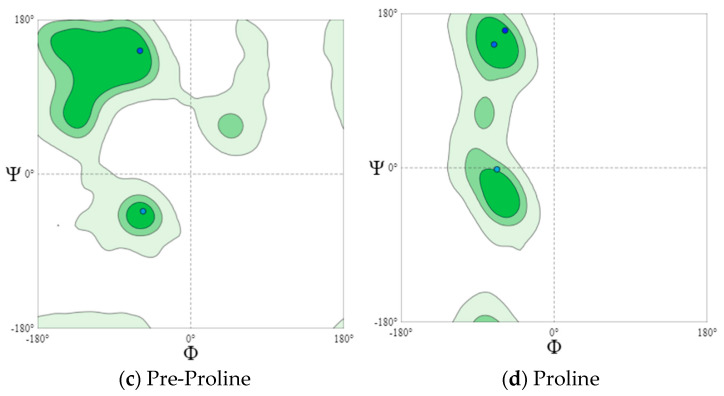
Ramachandran plots (Cheese A).

**Figure 5:**
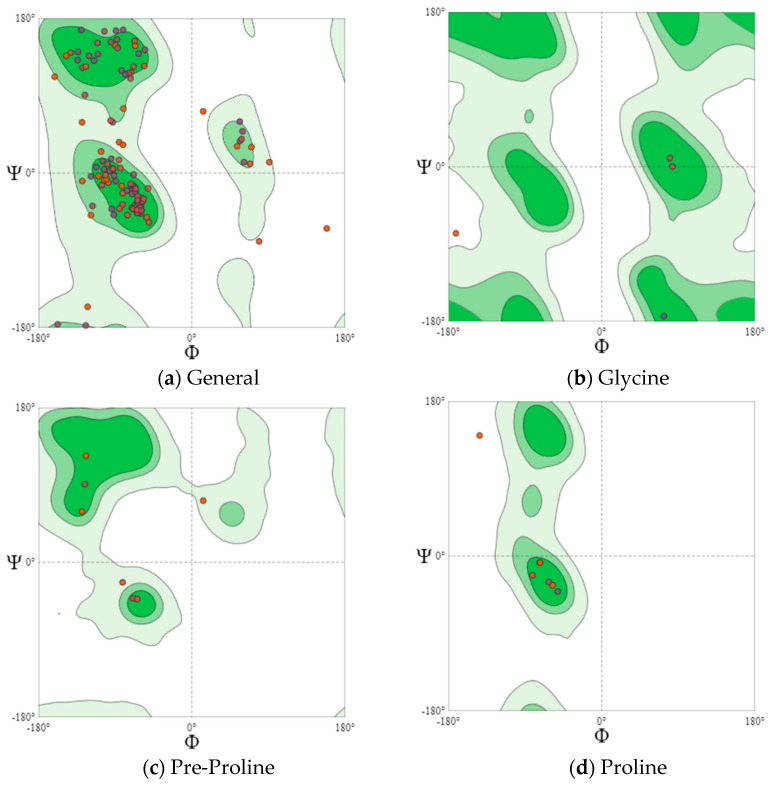
Ramachandran plots (Cheese B).

Eighteen potentially bioactive peptides out of 115 peptide sequences found in cheeses A and B are shown in Table 3. Their bioactivity ranged from 0.547239 to 0.870583 units. The results of the assessment of bioactive properties using the database of bioactive milk peptides MBPDB confirmed the biologically active properties of the identified peptide sequences. Further studies were carried out using the in silico method using the online resources presented in Section 2.6, Section 2.7, Section 2.8, Section 2.9 and Section 2.10. Further research featured the effect of the cultures on the protein structure. In Figure 4 and Figure 5 are the Ramachandran plots that visualize the dihedral angles of the polypeptide backbone (ψ and φ) in proteins.

Each point on the Ramachandran plots represents one amino acid. In a polypeptide, the backbone bonds rotate relatively freely. These rotations are represented by torsion angles Phi (φ) and Psi (ψ), respectively. The white areas correspond to conformations where the polypeptide atoms come closer than the sum of their van der Waals radii. These regions are sterically forbidden for all amino acids except glycine, which is unique in that it lacks a side chain. Glycine molecules were observed in both cheese samples. Dark green areas correspond to conformations without steric collisions, i.e., they are the regions allowed for the arrangement of amino acids, namely the α-helix and β-helix conformations. Light green areas show the regions that are allowed in case the van der Waals radii are slightly shorter, i.e., the atoms can move a little closer together. This additional region corresponds to the left α-helix.

L-amino acids cannot form extended sections of the left helix, but individual residues sometimes adopt this conformation. As a rule, they are represented by glycine, but they can also be asparagine or aspartate if the side chain forms a hydrogen bond with the main chain and, therefore, stabilizes this otherwise unfavorable conformation. The forbidden regions for amino acids in the model space are usually associated with the steric hindrance between the C-β-methylene group of the side chain and the atoms of the main chain. Glycine has no side chain and can take Phi (φ) and Psi (ψ) angles in all four quadrants of the Ramachandran plot. As a result, glycine is often found in the turning regions of proteins where any other residue would be sterically hindered.

The main areas are represented by the two dark green areas, while the three allowed areas are light green. The nuclear regions (dark green in Figure 4a and Figure 5a) represent the most favorable combinations of φ and ψ and contain the highest number of points. The allowed areas (light green) either cluster around the main areas or detach from the main area. Nevertheless, they contain fewer data points than the main areas (dark green). The white areas are prohibited for amino acids.

For a more detailed analysis, we modeled Ramachandran plots for glycine (B), pre-proline (C), and proline (D). These amino acids have different local steric hindrance properties and can take into account the effect of neighboring sequences. The proline side chain is covalently linked to the preceding N backbone. Thus, proline is more strictly prohibited than conventional residues. The residues, immediately before Pro (prePro), are limited as a result of the steric interaction with the proline ring. The other 16 types of amino acids prefer different regions, but their outer contours that separate the allowed regions from rogue ones match very closely. That is why they are all grouped together in the general case of distribution (Figure 4a and Figure 5a). The general Ramachandran plot shows that the main amino acids in cheese sample B are within the right α-helix, since the points representing the location of the amino acids in the figure are presented at the lower left border of the plot. The amino acids of cheese A are concentrated in the upper left border, which means that this sample has a left-handed β-helix.

**Figure 6:**
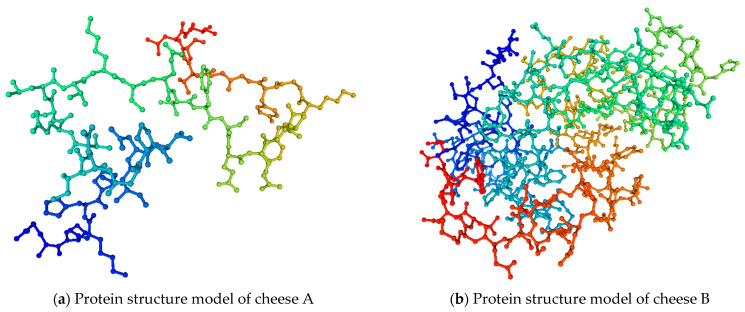
Protein structure models of cheese A and cheese B.

With the help of the SWISS-MODEL online service (https://swissmodel.expasy.org/interactive (accessed on 10 June 2022)), they developed the protein structure models of the studied cheeses (Figure 6).

**Figure 7:**
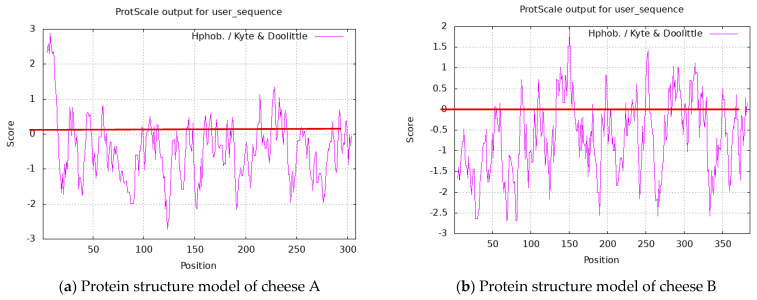
Topology of protein hydrophobicity and hydrophilicity.

Based on the obtained amino acid sequences, we determined the degree of hydrophilicity and hydrophobicity of proteins. Their graphs were built using the ProtScale online service based on the Kite and Doolittle scale (Figure 7).

In Figure 7a, it is illustrated that the peptides possess hydrophilic properties since most graph peaks on axis Y range from –2.8 to 0, which hints at the hydrophobic properties of the peptides on axis Y. Most hydrophobic regions have the following peptide sequences: TVDDKHYQKA and TESQSLTL. Most peaks are amino acids: aspartic acid, threonine, and serine. These amino acids have hydrophobic properties and 1–4 uncharged side radicals. Protein regions with such amino acid residues can hydrate and interact with other similar residues by hydrogen bonds. These peaks characterize the order of amino acids with hydrophobic properties. As for the control sample, the maximal value on the hydrophobicity scale belongs to threonine located in the 11-T region: it is 2.875 units. The highest hydrophilic properties belong to valine located in the 127-V region: it is 2.798.

Cheese B (Figure 7b) has the following characteristic trait. The main part of the peaks in the hydrophobic region is in regions 131–154 and 198–202 with peptides CSEKLDQW, LCEKL, HAQQKEPIM, and IPNPI Q, respectively. Most peaks above 0 are represented by amino acids Q, P, L, K, I, and E, which possess hydrophobic properties and 1–3 uncharged side radicals. Isoleucine (150—I) has a maximal hydrophobicity value of 2.00 units. Like in the control sample, the peptide sequences of the test sample have a predominantly hydrophilic nature.

The obtained data confirm the results published in [67,68] on the hydrophobic and hydrophilic properties of these amino acids.

The next research stage featured the characteristics of potential bioactive peptides. Their structure was compiled using online servers. The characteristics of bioactive peptides are presented in Table 4.

The assessment in Table 4 shows that the peptide samples are ACE inhibitors, DPP-4 inhibitors, and antioxidants. Based on the in silico studies, *Lc. lactis* subsp. *lactis* and *Lc. lactis* subsp. *cremoris* in the control sample had a greater effect on the development of bioactive peptides. Thus, the control sample contained thirteen bioactive peptide sequences, while the experimental sample with *Lac. casei* had only seven.

**Figure 8:**
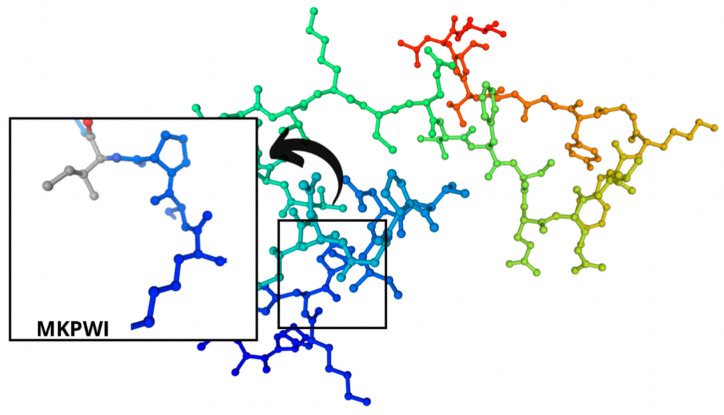
Regions in the structure of bioactive peptides, cheese A. The arrows point to the location of the peptide sequence in the protein molecule structure.

**Figure 9:**
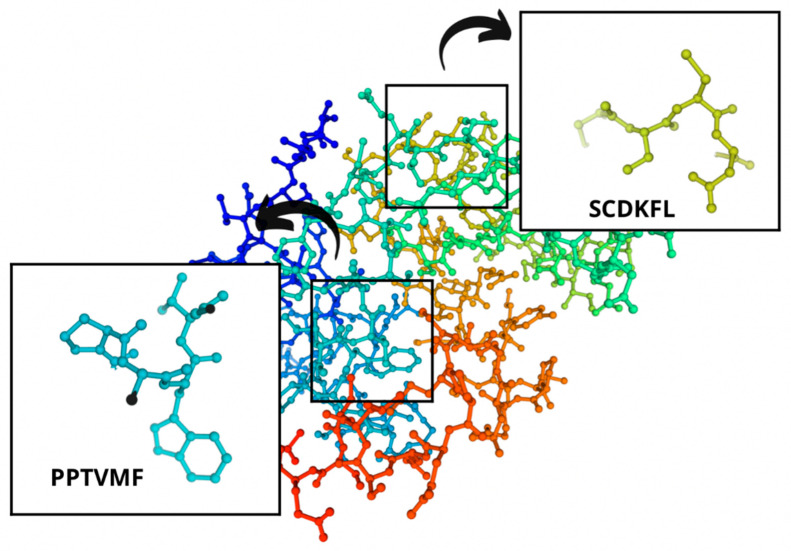
Regions in the structure of bioactive peptides, cheese B. The arrows indicate the location of the peptide sequence in the protein molecule structure.

The sections of the bioactive peptides in cheeses A and B are shown in Figure 8 and Figure 9.

The identified bioactive peptides had aromatic rings that were represented mainly by tryptophan, tyrosine, and phenylalanine. As for the peptide bioactivity in Table 3, peptide sequence SCDKFL with high peptide bioactivity 0.8444337 was found only in cheese B with *Lac. casei*. Peptide sequences MKPWI (0.853622) and PSGAW (0.870583) were found both in the control cheese A and the experimental cheese B. These sequences also demonstrated a high bioactivity of peptides. All these peptide sequences included such essential amino acids as lysine, isoleucine, leucine, phenylalanine, methionine, and tryptophan, which are beneficial for human health and must be included in the diet. All these sequences inhibit the angiotensin-converting enzyme (ACE). ACE inhibitors are responsible for multifactorial actions in the human body, e.g., they relax blood vessels, thus, reducing blood pressure. In medical practice, ACE inhibitors are known to reduce the hospitalization incidence for heart failure as they increase life expectancy, exercise tolerance, and life quality [1,24,69].

## 4. Conclusions

Bioactive peptides appear as a result of biochemical and microbiological reactions under the action of proteolytic enzymes and microorganisms in starter cultures during the cheese ripening. They can act as ACE inhibitors. Regular consumption of mature cheeses not only satisfies the need for protein and essential amino acids but also, under certain circumstances, makes it possible to reduce or avoid taking pharmacological drugs. This issue, however, requires further research in cooperation with medical scientists. The authors believe that cheese can be regarded as a functional product. Cheese has a long shelf life: the longer the period of its ripening, the more bioactive peptides and amino acids they accumulate. This research proves that the accumulation of bioactive peptides in different cheeses can be predicted depending on the strain of microorganisms in their starter cultures. Biopeptide studies open certain prospects because various strains of microorganisms used in the food industry, including cheese-making, are so beneficial that they can potentially replace some pharmacological preparations, thus realizing the health-via-food concept. Further research will feature different parameters and raw materials in cheese formulations.

## Figures and Tables

**Table 2:** Chemical composition of model cheeses on day 90.

Index	Cheese A * (Control)	Cheese B ** (Test)
Mass fraction of solids, %	55.16 ± 0.21	54.85 ± 0.23
Mass fraction of fat in solids, %	48.93 ± 1.95	49.50 ± 1.04
Mass fraction of total protein, %	20.23 ± 0.28	21.23 ± 0.31
Mass fraction of salt, %	2.34 ± 0.72	3.13 ± 0.55
pH	4.74 ± 0.18	4.13 ± 0.06

Cheese A * contained starter cultures of *Lc. lactis* subsp. *lactis* and *Lc. lactis* subsp. *cremoris.* Cheese B ** contained starter cultures of *Lc. lactis* subsp. *lactis*, *Lc. lactis* subsp. *cremoris* and *Lac. casei*, 0.5% + 0.5%.

**Table 3:** Peptide sequences and their bioactivity.

Sample	Fragment in Amino Acid Sequence	Peptide Sequence in one Letter Code *	Bioactivity	Molecular Weight, Da
A	1–5	MMKSF	**0.730558**	643.3
A	1–5	MKVLI	0.194789	603.4
A	1–7	MMSFVSL	0.488188	894.4
A	7–11	LVVTI	0.0498343	624.3
A	8–12	VVTIL	0.0759513	624.3
A	11–16	TCGAQA	0.162151	550.2
A	16–21	RPKHPI	0.396125	747.5
B	20–28	EQLTKCEVF	0.154299	1176.5
B	22–31	KHQGLPQEVL	0.224327	1148.6
A	22–31	NVPGEIVESL	0.118838	1056.6
A	23–28	TKCEVF	0.182253	806.3
A, B	31–35	SQETY	0.0993374	627.3
B	31–42	SQETYKQEKNMA	0.127054	1536.6
A	32–38	SSSEESI	0.0916819	978.2
A, B	35–42	KGYGGVSL	0.226416	780.4
A, B	38–45	GGVSLPEW	**0.628953**	844.4
A, B	39–43	FSDKI	0.403109	609.3
A	39–45	TRINKKI	0.0758366	872.6
A	43–50	INPSKENL	0.208794	914.5
A	43–50	PEWVCTTF	**0.703949**	1142.4
A	44–50	NPSKENL	0.209493	801.4
A, B	46–50	VCTTF	0.383259	650.2
A	48–59	GKEKVNELSKDI	0.105419	1439.7
A, B	51–62	CSTFCKEVVRNA	0.32156	1436.6
A	51–55	QSAPL	0.491633	515.3
A, B	51–55	HTSGY	0.16794	724.2
A, B	51–59	HTSGYDTQA	0.090863	1139.3
A	52–56	VLSRY	0.181831	637.4
A, B	54–59	SRYPSY	0.442889	1012.3
A, B	55–67	CKEVVRNANEEEY	0.0499419	1662.7
A	56–60	DTQAI	0.094131	547.3
A	57–61	PSYGL	**0.748397**	696.2
B	60–67	GSESTEDQAMEDI	0.0764591	1571.5
A, B	60–68	GSESTEDQA	0.0703232	1163.3
B	61–69	VQNNDSTEY	0.0623822	1149.4
B	63–69	NEEEYSI	0.0502548	883.4
A	64–70	YQQKPVA	0.12662	913.4
A	65–70	QQKPVA	0.121875	670.4
A	69–73	QTQSL	0.105763	656.3
B	70–77	GSSSEESA	0.0857066	833.3
A	72–76	INNQF	0.436729	635.3
A	73–77	NNQFL	**0.693175**	635.3
B	73–80	KQMEAESI	0.0604811	1015.4
A	76–81	TFPGPIP	**0.774112**	627.4
A	78–86	EVATEEVKI	0.0379171	1017.5
B	78–86	ESISSSEEI	0.0703352	1140.4
B	80–91	CKDDQNPHSSNI	0.313549	1437.5
B	80–94	CKDDQNPHSSNICNI	**0.61108**	1687.7
B	87–93	TVDDKHY	0.0687443	957.4
A	87–96	TVDDKHYQKA	0.0839258	1284.6
B	87–96	VPNSVEQKHI	0.119726	1150.6
A, B	87–92	AVRSPA	0.171253	680.3
A, B	88–94	VRSPAQI	0.149257	850.4
A, B	90–94	EKTKI	0.0411874	618.4
A, B	90–96	EKTKIPA	0.0778473	866.4
A, B	93–102	TQTPVVVPPF	0.213655	1164.6
B	93–103	TQTPVVVPPFL	0.336248	1197.7
B	95–100	SCDKFL	**0.844337**	712.3
A	96–100	QWQVL	**0.584037**	673.4
A	100–104	LDDDL	0.294973	590.3
A	101–108	DDDLTDDI	0.139488	921.4
A	103–109	LNENKVL	0.0983577	829.5
A	104–109	NENKVL	0.0944044	716.4
B	107–117	KSCQAQPTTMA	0.451658	1325.5
A	110–115	VLDTDY	0.109603	805.3
A, B	115–119	RLKKY	0.162844	707.5
A	116–122	DKVGINY	0.13778	644.4
A, B	120–126	KVPQLEI	0.109796	826.5
A	122–127	CMENSA	0.152971	734.2
A, B	125–131	DQVKRNA	0.0817682	830.4
A	127–131	VPNSA	0.12024	567.2
A	127–134	PKYPVEPF	**0.630562**	976.5
B	128–133	EPEQSL	0.108742	702.3
A	128–134	EPEQSLA	0.111383	853.3
B	129–134	LCSEKL	0.227722	888.4
A, B	130–134	CSEKL	0.263366	692.4
A	130–134	PVEPF	**0.604782**	588.3
B	130–137	CSEKLDQW	0.393924	579.3
A, B	135–140	TESQSL	0.0615565	904.2
A, B	135–142	TESQSLTL	0.0988356	1198.3
A	135–143	TPTLNREQL	0.0748829	1151.5
B	136–142	HSMKEGI	0.177846	801.4
A, B	138–142	LCEKL	0.274007	1008.4
A, B	143–151	HAQQKEPIM	0.258844	1081.5
A	148–159	SGEPTSTPTTEA	0.0824745	1177.5
A	160–165	VESTVA	0.0342661	605.3
A, B	160–167	VESTVATL	0.0738447	899.4
A, B	163–176	TKKTKLTEEEKNRL	0.0531694	922.5
B	166–172	SFNPTQL	**0.619614**	806.4
A	166–174	TLEDSPEVI	0.0723477	1162.4
A, B	167–172	PPTVMF	**0.826762**	771.3
A	168–180	EDSPEVIESPPEI	0.166577	1440.7
A	168–172	NPTQL	0.293133	572.3
A, B	173–180	PPQSVLSL	0.487707	840.5
A	174–178	YPSGA	0.433892	574.2
A, B	175–179	PSGAW	**0.870583**	597.2
A	175–180	ESPPEI	0.211756	671.3
A, B	179–186	SLSQSKVL	0.184348	941.5
A	180–186	KKISQRY	0.127085	922.5
A, B	181–186	SQSKVL	0.210141	741.4
B	181–189	TVQVTSTAV	0.0291752	1160.4
A, B	181–192	SQPKVLPVPQKA	0.343103	1281.8
A	182–188	VPLGTQY	0.161862	937.3
A, B	183–189	SQRYQKF	**0.584592**	1036.5
A, B	185–191	GTQYTDA	0.086917	755.3
A	189–194	TDAPSF	0.417727	717.2
A	192–197	PSFSDI	**0.547239**	665.3
A, B	195–201	SDIPNPI	**0.573163**	755.4
A	200–204	QHQKA	0.0976463	611.3
A, B	202–213	IGSENSEKTTMPLW	0.351255	1533.5
A, B	205–209	MKPWI	**0.853622**	674.4
A	208–213	YQEPVL	0.234191	748.4
A	209–216	IQPKTKVI	0.108562	926.6
B	210–218	QPKTKVIPY	0.213985	697.4
A, B	217–221	PYVRY	0.446831	1233.6

* A—alanine; C—cysteine; D—aspartic acid; E—glutamic acid; F—phenylalanine; G—glycine; H—histidine; I—isoleucine; K—lysine; L—leucine; M—methionine; N—asparagine; P—proline; Q—glutamine; R—arginine; S—serine; T—threonine; V—valine; W—tryptophan; Y—tyrosine; Bold font indicates potential bioactive peptides

**Table 4:** Potential bioactive peptides.

Sample	Peptide Sequence	Bioactivity *	Potential Bioactive Properties **	Peptide Structure ***
A, B	PSGAW	0.870583	ACE inhibitors	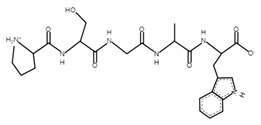
A, B	MKPWI	0.853622	ACE inhibitors	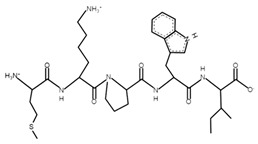
B	SCDKFL	0.844337	ACE inhibitor	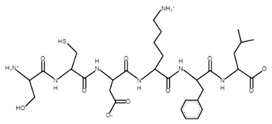
A, B	PPTVMF	0.826762	DPP-4 inhibitors	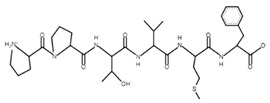
A	TFPGPIP	0.774112	DPP-4 inhibitor	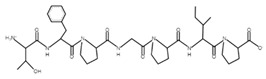
A	PSYGL	0.748397	Opioid	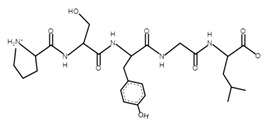
A	MMKSF	0.730558	ACE inhibitor	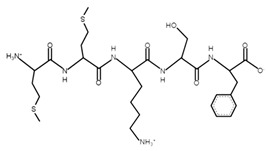
A	PEWVCTTF	0.703949	ACE inhibitor	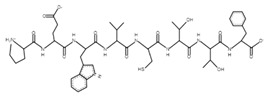
A	NNQFL	0.693175	DPP-4 inhibitor	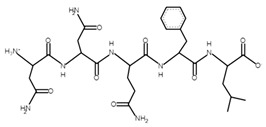
A	PKYPVEPF	0.630562	Antioxidant	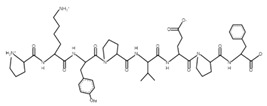
A, B	GGVSLPEW	0.628953	ACE inhibitors	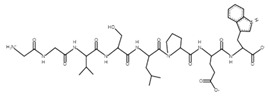
B	SFNPTQL	0.619614	ACE inhibitor	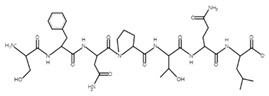
B	CKDDQNPHSSNICNI	0.61108	Antimicrobial	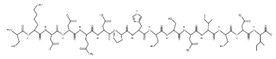
A	PVEPF	0.604782	Opioid; DPP-4 inhibitor; antioxidant	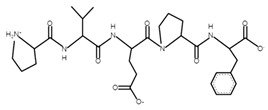
A	QWQVL	0.584037	Immunomodulating	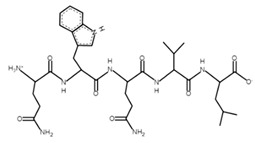
A, B	SDIPNPI	0.573163	Growth stimulators	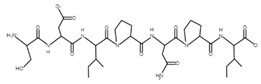
A	PSFSDI	0.547239	Anticancer; antimicrobial	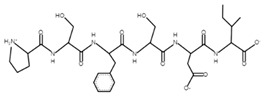

* The in silico study was based on the PeptideRanker online service; ** according to the MBPDB database of bioactive milk peptides; *** the modeling was based on the PepDraw online service.

## Data Availability

Data are available from the authors on request.
